# Teleradiotherapy Network: Applications and Feasibility for Providing Cost-Effective Comprehensive Radiotherapy Care in Low- and Middle-Income Group Countries for Cancer Patients

**DOI:** 10.1089/tmj.2014.0154

**Published:** 2015-07-01

**Authors:** Niloy Ranjan Datta, Michael Heuser, Massoud Samiei, Ragesh Shah, Gerd Lutters, Stephan Bodis

**Affiliations:** ^1^ Centre for Radiation Oncology, KSA-KSB, Kantonsspital Aarau, Aarau, Switzerland.; ^2^ Formerly, PACT Section, International Atomic Energy Agency, Vienna, Austria.; ^3^ Online Telemedicine Research Institute, Ahmedabad, India.; ^4^ Department of Radiation Oncology, University Hospital Zurich, Zurich, Switzerland.

**Keywords:** *teleoncology*, *telemedicine*, *telehealth*, *e-health*, *education*

## Abstract

Globally, new cancer cases will rise by 57% within the next two decades, with the majority in the low- and middle-income countries (LMICs). Consequently, a steep increase of about 40% in cancer deaths is expected there, mainly because of lack of treatment facilities, especially radiotherapy. Radiotherapy is required for more than 50% of patients, but the capital cost for equipment often deters establishment of such facilities in LMICs. Presently, of the 139 LMICs, 55 do not even have a radiotherapy facility, whereas the remaining 84 have a deficit of 61.4% of their required radiotherapy units. Networking between centers could enhance the effectiveness and reach of existing radiotherapy in LMICs. A teleradiotherapy network could enable centers to share and optimally utilize their resources, both infrastructure and staffing. This could be in the form of a three-tier radiotherapy service consisting of primary, secondary, and tertiary radiotherapy centers interlinked through a network. The concept has been adopted in some LMICs and could also be used as a “service provider model,” thereby reducing the investments to set up such a network. Teleradiotherapy networks could be a part of the multipronged approach to address the enormous gap in radiotherapy services in a cost-effective manner and to support better accessibility to radiotherapy facilities, especially for LMICs.

## Introduction

The rapid strides in computer technology, aided by concurrent developments in modern telecommunications and information technology, have enabled the emergence of telemedicine as a very potent tool in modern healthcare. Telemedicine and e-health are thus being explored by health providers in a growing number of medical specialties, including oncology, to enable wider coverage of health services and also to improve health economics. This assumes significance for resource constraint countries, especially the low- and middle-income group countries (LMICs).

According to the World Cancer Report 2014, published by the International Agency for Cancer Research of the World Health Organization, globally cancer remains a major cause of morbidity and mortality, with 14 million new cancer cases and 8 million cancer-related deaths reported in 2012.^1^ Sixty percent of these new cancer cases are in Africa, Asia, and Central and South America, with 70% of the cancer deaths being accounted from these regions. According to the World Health Organization, the cancer incidence between 2008 and 2030 is projected to rise by 82%, 70%, and 58% in the low, low-middle, and upper-middle income groups of countries, respectively, compared with 40% in high-income countries (HICs). Moreover, two-thirds of the cases are expected in LMICs.^2^ The present situation for cancer care in LMICs and the challenge of making radiotherapy, a key component of cancer management, accessible in these countries have been major concerns recognized by all the stakeholders both at country levels and also internationally.^3,4^

## Gap in Access to Radiotherapy in LMICs

**Fig. 1.**
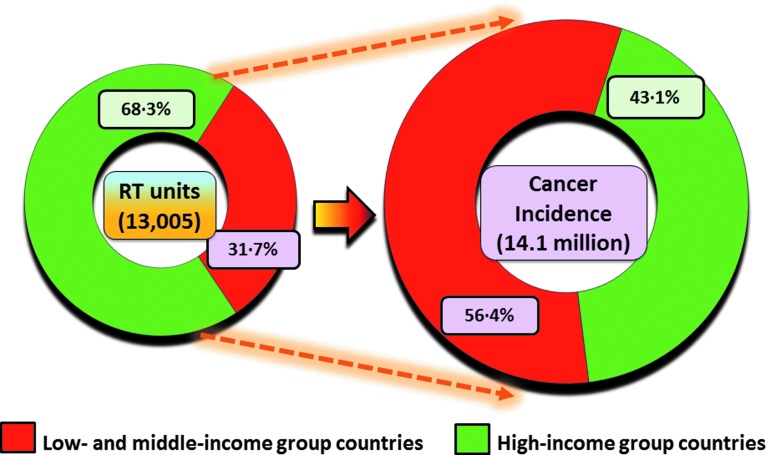
Available teletherapy units and the proportion of cancer incidence in low- and middle-income group countries versus high-income countries. Fifty-seven percent of cancer patients are estimated to be in low- and middle-income group countries but have access to only 32% of total available teletherapy units globally. Color images available online at www.liebertpub.com/tmj

Presently, of the 13,002 radiotherapy units that are in use globally, only 32% of these are available to cancer patients from LMICs, which account for 57% of the global cancer cases (*Fig. 1*). Radiation therapy is estimated to be required in 45–55% of newly diagnosed cases.^5^ Of those cured, 40% are by radiotherapy alone or in combination with other modalities.^6^ The 66th United Nations General Assembly has listed cancer as a part of “a rising epidemic” of the noncommunicable diseases and has noted the inadequate radiotherapy services in developing countries.^7^

**Fig. 2.**
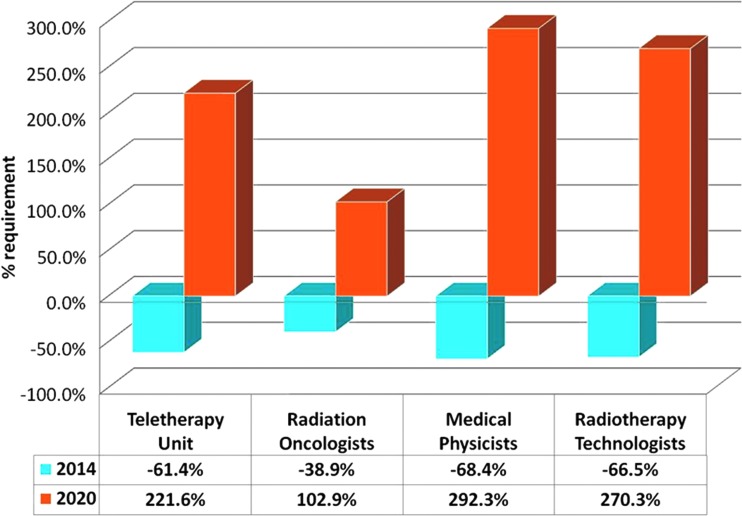
Present deficit in teletherapy units, radiation oncologists, medical physicists, and radiotherapy technologists in low- and middle-income countries and the additional requirements by 2020 for each of these radiotherapy capacity components. Color images available online at www.liebertpub.com/tmj

In a recent assessment of the radiotherapy infrastructure and staffing in the 139 LMICs, it has been reported that only 4 of the 139 LMICs have the requisite number of radiotherapy units.^8^ Fifty-five of these countries (39.5%) have no radiotherapy facilities at present. Patient access to radiotherapy in the remaining 80 LMICs ranges from 2.3% to 98.8% (median, 36.7%). By 2020, these 84 LMICs would additionally need 9,169 radiotherapy units, 12,149 radiation oncologists, 9,915 medical physicists, and 29,140 radiotherapy technologists. Moreover, *de novo* radiotherapy facilities would have to be considered for those with no services^8^ (*Fig. 2*).

Because the setting up of radiotherapy facilities involves significant capital investment, it might be a difficult task for most resource-constrained LMICs. Moreover, because highly specialized staffing of radiation oncologists, medical physicists, and radiotherapy technologists for a safe practice and execution of the various radiation oncology techniques is needed, the shortage of trained human resources also adds to the gaps in radiotherapy accessibility. It is thus necessary to effectively utilize the limited resources to meet the projected requirements and find ways to offer “quality assured radiation therapy” to the patients. In 2003, the International Atomic Energy Agency estimated that over a decade, some USD 2.5 billion would be needed to provide adequate radiotherapy services in developing countries, half of which would be for treatment machines and the rest for training professionals for safe and effective radiotherapy practices.^9^ Today, this amount would have certainly increased as there exists considerable gaps in radiotherapy accessibility, and the requirements would grow significantly by 2020.

Telemedicine can be an effective tool to help bridge this gap. This has been effectively explored by several countries—both HICs and LMICs—with very encouraging results, as is evident from various reports.^10–16^ In most of these reports, telemedicine applications have been restricted to teleconsultation, teleradiology, telepathology, and tele-education. In 2000, Olsen et al.^15^ examined the feasibility and requirements of telemedicine in radiotherapy treatment planning. This concept has been further extended here to propose creating an integrated three-tier radiotherapy service consisting of primary, secondary, and tertiary radiotherapy centers (PRTCs, SRTCs, and TRTCs, respectively) in developing countries using a teleradiotherapy network. This could be cost-effective, help bridge the gap, and give a larger proportion of patient access to the state-of-the-art technology in radiation therapy.^17^

## Three-Tier Teleradiotherapy Network for LMICs

**Fig. 3.**
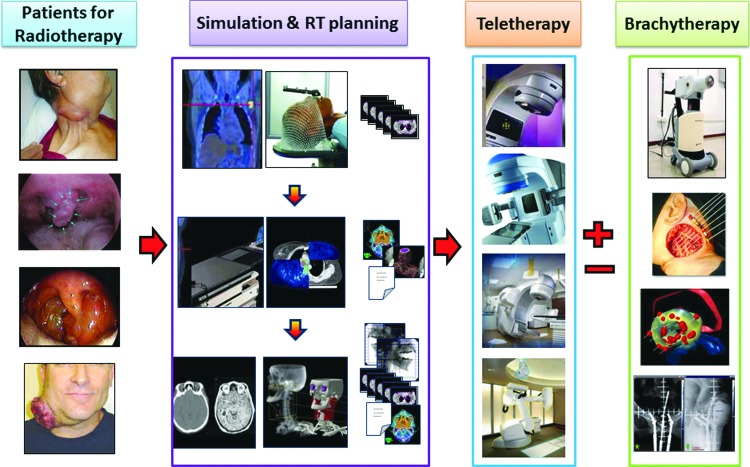
A schematic representation of the radiation therapy (RT) treatment process. All patients for RT would undergo treatment simulation and dose computation using treatment planning. This would be followed by teletherapy, which could range from conventional to the state-of-the-art treatment techniques. Some of these patients might also be treated with brachytherapy either alone or integrated with teletherapy in a planned manner, depending on tumor types and stage. Color images available online at www.liebertpub.com/tmj

Radiation therapy undergoes a sequence of processes involving patient treatment simulation with either a conventional simulator or computed tomography-based simulation, target (tumor) and normal structure delineation, and dose calculations using a treatment planning system before treatment execution using a radiotherapy unit (*Fig. 3*). Depending on the tumor site and stage, some of the patients could be treated either using brachytherapy alone or a combination of radiotherapy and brachytherapy. Thus, a radiotherapy center should be equipped with all these equipment for carrying out treatment using radiotherapy and brachytherapy.

Radiation therapy could be used either as a sole modality of treatment for curative purpose (radical radiotherapy) or in combination with surgery (pre- or postoperative radiotherapy) or in combination with chemotherapy. In some situations, all the three treatment modalities—namely, radiotherapy, surgery, and chemotherapy—could be advocated depending on the tumor type, its stage, the patient's general condition, and departmental treatment policies. In patients with advanced disease conditions (e.g., painful bony metastasis, spinal cord compressions), a short course of palliative radiotherapy could be used for symptomatic relief of symptoms.

The 10 most common cancers in both sexes in less developed regions of the world are lung, breast, colorectum, prostate, stomach, liver, uterine cervix, esophagus, urinary bladder, and non-Hodgkin's lymphoma.^18^ All of these need radiotherapy either as a primary treatment modality or in combination with other modalities like surgery or chemotherapy. Thus, radiotherapy constitutes a key modality in the modern multimodality management of cancers, and therefore it becomes imperative to have adequate radiotherapy facilities for any cancer treatment center.

**Fig. 4.**
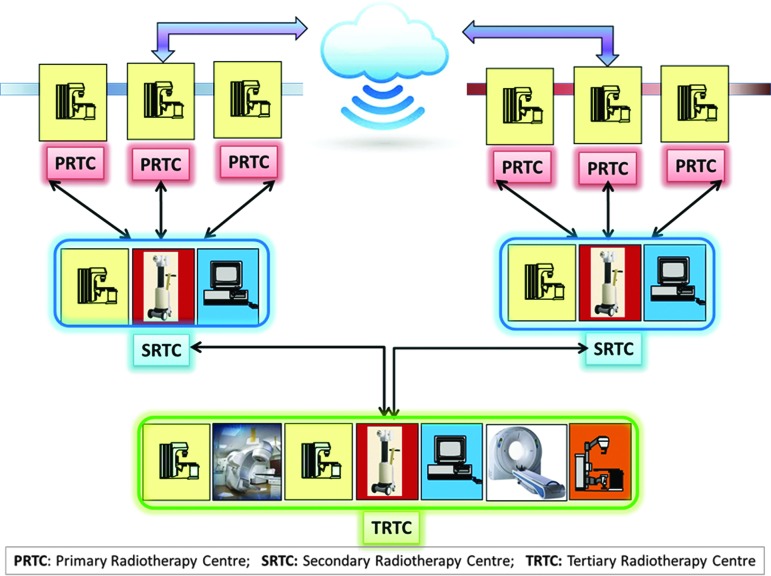
A schematic representation of a three-tier teleradiotherapy network among primary, secondary, and tertiary radiotherapy centers (PRTC, SRTC, and TRTC, respectively). Color images available online at www.liebertpub.com/tmj

**Fig. 5.**
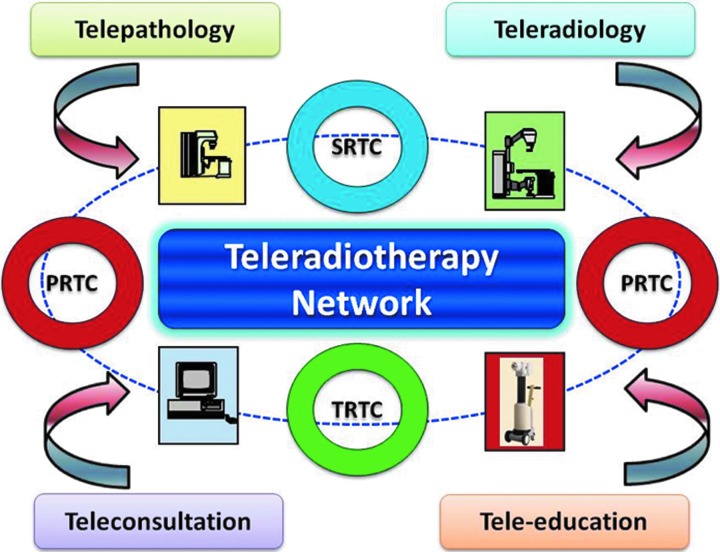
An integrated teleoncology concept with teleradiotherapy, telepathology, teleradiology, teleconsultation, and tele-education. PRTC, primary radiotherapy center; SRTC, secondary radiotherapy center; TRTC, tertiary radiotherapy center. Color images available online at www.liebertpub.com/tmj

Datta and Rajasekar^17^ proposed a three-tier system consisting of a PRTC (primarily radiotherapy center), an SRTC (secondary radiotherapy center), and a TRTC (tertiary radiotherapy center), linked with a teleradiotherapy network (*Fig. 4*). The various components of this network have been described in an earlier publication.^17^ In brief, it would consist of:

a. *PRTC.* These centers could just have a radiotherapy unit and be able to act as a center for delivery of radiotherapy. They can cater to the needs of a population of around 2–4 million. Treatment planning and simulation would have to be carried out at the next higher-level center. Such a PRTC would normally be located close to patients' homes, which would save both money and time for them to travel to far-reaching places where they are often required to stay for the total duration of treatment, lasting around 5–7 weeks. The PRTC could be the focal point for cancer prevention and education program at the grass root level and also be responsible for organizing early detection and other screening programs.
b. *SRTC.* The SRTC could be an existing radiotherapy center, which could have the basic requirements of a radiotherapy center consisting of radiotherapy, brachytherapy, and treatment planning systems. These centers should be able to carry out the simulation and treatment planning of patients referred from the PRTC, apart from those who directly attend these SRTCs. Patients could then be sent back to the PRTC for radiotherapy delivery. The SRTC should also coordinate the activities of various PRTCs linked with them and provide technical help and expertise. In clinical situations, where patients need an advanced radiotherapy treatment facility, they could be referred to the TRTC.
c. *TRTC.* The TRTC could be a center of excellence, having state-of-the-art technology—namely, three-dimensional conformal, intensity-modulated radiotherapy, stereotactic radiotherapy, stereotactic radiosurgery, and advanced brachytherapy techniques. The TRTC could be located at a tertiary-care teaching hospital with proper infrastructure and also support services. A country could have one or several of these TRTCs distributed evenly in accordance with the country's population density and available resources. The TRTC could act as a referral center for both SRTCs and PRTCs, coordinate activities of PRTCs and SRTCs, and be responsible for teaching and training of the human resources at these subsidiary centers. The TRTC would also be involved in formulating various research protocols and trials, both clinical and translational, based on the needs and problems of the particular geographical area.
d. *Linking PRTCs, SRTCs, and TRTCs through a teleradiotherapy network.* All the three levels of radiotherapy centers could be integrated to facilitate the clinical, teaching, quality assurance, and research activities of these centers. Moreover, because in radiotherapy most of the images are compatible with Digital Imaging and Communications in Medicine (DICOM), its radiotherapy extension (DICOM-RT), and Health Level-7, effective exchanges among these centers could be seamlessly integrated through the network. Other telemedicine activities, like telepathology, teleradiology, teleconsultation (with multidisciplinary tumor boards), and tele-education through a virtual classroom concept, could be included as well to create an integrated tele-oncology network (*Fig. 5*). The TRTC could be considered as the primary hub, and terminals at PRTCs and SRTCs could constitute the secondary hubs linked through either the Integrated Services Digital Network or satellite or cloud computing.

For health providers, this proposal could lead to reduction of operating costs through centralization and optimization of resources and reduction in costs of training and updating skills of the technical staff and physicians without any travel and absence from their place of work. This could lead to significant tangible benefits.^19^

For the successful implementation of the teleradiotherapy network as discussed above, the requirements would be nearly similar to any other programs related to telemedicine. However, because the applications related to radiotherapy treatment planning and execution involve storage and transfer of large volumes of data, the linked sites should be able to have a common infrastructure for the maximum use of synergies with high-speed Internet connections. All necessary data and applications should be stored on centralized servers and be available at both locations in real time. The solution needs to be fail-safe and ensure a frictionless, paperless clinical workflow between the linked sites. Moreover, the infrastructure should be scalable for connecting additional sites in the near future. Apart from these, it needs adequate training of staff, their willingness to adapt to the technology, updating and maintaining the equipment with adequate antivirus measures, and, perhaps most importantly, a mutual willingness and trust with confidence building between the staff members of the various centers. Finally, the policy makers and stakeholders at all institution and government levels, particularly those involved in national cancer control planning, should be made fully aware of the utility and application of the telemedicine technology to maintain long-term sustenance through their commitment and support.

## Teleradiotherapy Network in HICs

The teleradiotherapy network has also found acceptance in many HICs. British Columbia launched a tele-oncology project in December 2008. The primary objective was to improve access for patients and their families in remote and rural areas of the province to specialized oncology service provided by the regional cancer centers and to provide specialized telehealth and videoconferencing in their cancer centers. This could also improve the oncology-related education for care providers.^20^ Norway has also an established telemedicine network, and the Norwegian Radium Hospital has linked its radiotherapy service to two satellite hospitals several hundred kilometers away.^15,21^ Similar endeavors have been undertaken by centers in Japan^22^ and Germany.^23^

The Centre for Radiation Oncology at Kantonsspital Aarau (KSA) is one of the five top radiation oncology centers in Switzerland. The Centre has recently established a satellite radiation oncology center at Kantonsspital Baden (KSB) within the Canton Aargau and proposes to link the center with a telemedicine network, especially designed for radiation oncology purposes. In the process of modernization, three new state-of-the-art radiotherapy units would be installed at KSA, whereas the fourth unit, placed at KSB, would be provided with the oncology information system for patient scheduling, electronic patient record, DICOM imaging data and recording and verification system, and radiotherapy treatment planning system from a common data center.

**Fig. 6.**
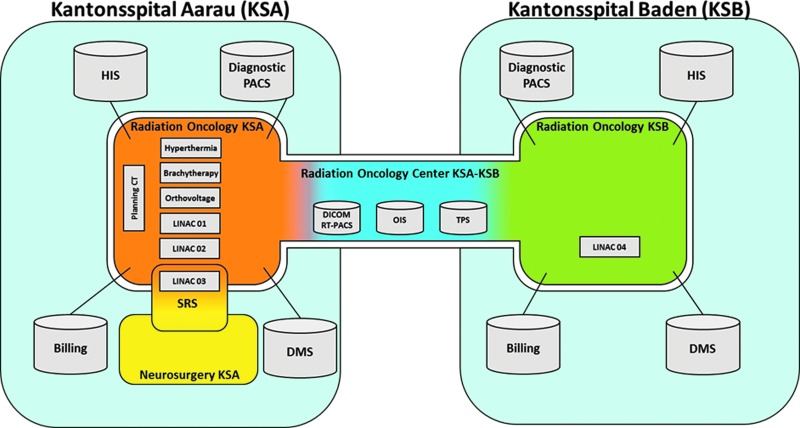
Central information technology providing service to different sites for the Centre of Radiation Oncology Kantonsspital Aarau (KSA) and Kantonsspital Baden (KSB), Switzerland. CT, computed tomography; DICOM RT, radiotherapy extension of Digital Imaging and Communications in Medicine; HIS, health information system; LINAC, linear particle accelerator; OIS, oncology information system; PACS, Picture Archiving and Communication System; TPS, treatment planning system; DMS, data management system; SRS, stereotactic radiosurgery. Color images available online at www.liebertpub.com/tmj

**Fig. 7.**
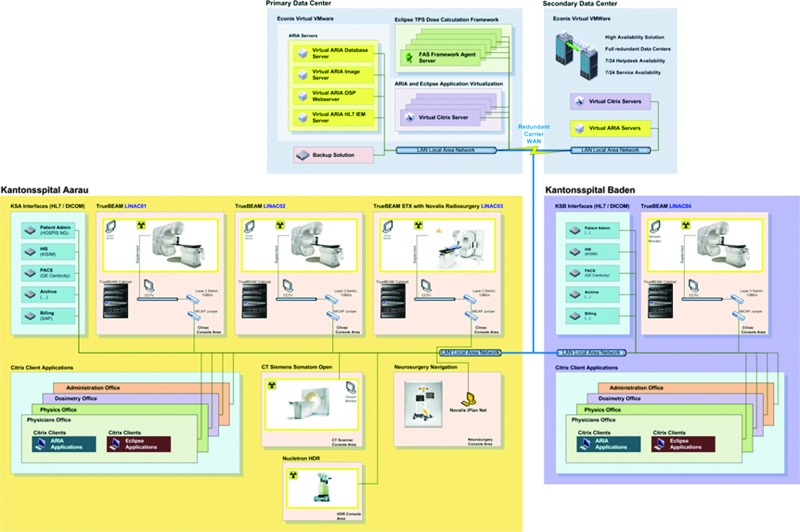
Information technology network schema for the Centre of Radiation Oncology, Kantonsspital Aarau and Kantonsspital Baden, Switzerland. Color images available online at www.liebertpub.com/tmj

With the exception of stereotactic treatments, which will only be offered only at KSA, patients will be able to obtain all other treatments at either location, thus maximizing patient comfort. The treatment planning for complex treatment plans (intensity-modulated radiotherapy, volumetric-modulated arc therapy, stereotactic body radiotherapy, and stereotactic radiosurgery) will be performed centrally at KSA, whereas simple treatment planning (two-dimensional, three-dimensional) will be undertaken directly at the respective location at KSA or KSB. The location at KSB can thus be operated with reduced staff. The case discussions and presentations of the treatment plans will be carried out daily between the two sites and also in interdisciplinary periodic virtual tumor boards via an online collaboration platform (Cisco [San Jose, CA] WebEx™). The goal would be the realization of the software modules in a Software-as-a-Service concept in collaboration with the medical device manufacturers. The associated information technology infrastructure would be implemented as a cloud-hosted solution. In addition, both sites would work independently on a common infrastructure for the maximum use of synergies. All necessary data and applications for the radiotherapeutic treatment would be stored on centralized servers and made available at both locations in real time. The solution would be fail-safe and highly available to ensure a frictionless paperless clinical workflow of the two sites (*Figs. 6* and *7*).

The hardware infrastructure would have at least two data centers in Switzerland, in order to guarantee data retention and data protection according to Swiss regulations. The data would be stored on modern storage systems and in addition secured by means of backup to disk, stored to a second data center. In case of a disaster, immediately failover procedures will be initiated to redundant systems for an uninterrupted treatment. The individual software applications are deployed hardware independent via Citrix^®^ (Santa Clara, CA), therefore allowing the work of location-independent mobile devices. The network connection to the data center is realized with a guaranteed bandwidth of 50 megabits/s as a Layer 3 virtual private network, with redundant feeds from the data center to each individual site. The infrastructure would be monitored 24 h, 7 days a week.

The network would enable sharing of infrastructure and the reduction of costs for both partners. Joint use of shared resources would lead to a reduction of capacity requirements, thereby reducing the overhead and costs of all cooperation partners compared with providing the full infrastructure for each site. This ensures increased service quality in terms of reliability and performance, as it would not be possible otherwise for smaller sites to realize this individually. The hosted private cloud solution is secured and only available for cooperating clinics with a dedicated wide area network connection. Moreover, the advantage of the cloud solution would permit very easy scalability. In principle, a centralized hosting of dedicated software modules for radiation therapy centers at the national or international level is possible. This is technically feasible with increasing number of participants; thereby, the cost for each participant would be reduced. Varian Medical Systems (Palo Alto, CA) is associated with this project. It is expected that such a model would help LMICs share their existing resources in a most cost-effective manner, thereby saving infrastructure costs and at the same time providing better care in the periphery within the limited resources available.

## Teleradiotherapy Network in LMICs

The teleradiotherapy network proposal as discussed above could be adopted in various LMICs, depending on their existing infrastructure and resource availability for both infrastructure and staffing. As a specific example, the proposal as it has been applied in India is described below.

**Fig. 8.**
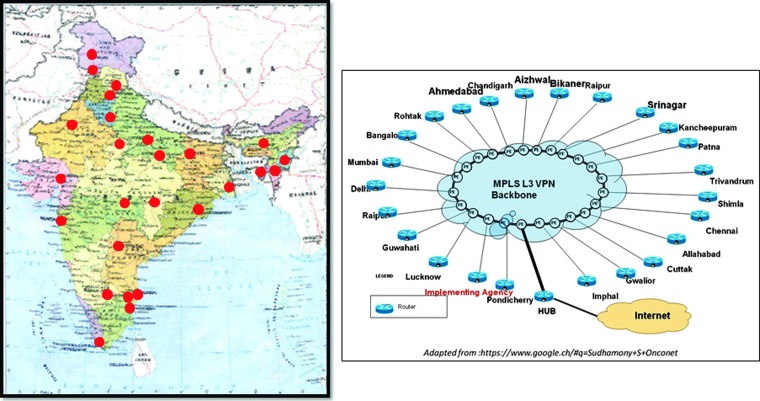
Regional cancer centers and peripheral centers in India are to be linked under the OncoNET (National Cancer Control Programme, Ministry of Health, Government of India; www.mohfw.nic.in/index1.php?lang=1&level=2&sublinkid=323&lid=323) with the overall architecture of the OncoNET network. MPLS L3 VPN, multiprotocol label switching Layer 3 virtual private network. Adapted from https://www.google.ch/#q=Sudhamony+S+Onconet. Color images available online at www.liebertpub.com/tmj

India presently has one of the highest infrastructures and staffing of the 139 LMICs. However, with a burden of more than 1 million cancer patients, the radiotherapy accessibility with even over 500 radiotherapy units is only around 36.3%.^8^ Presently it has a deficit of 899 radiotherapy units, 2,186 radiation oncologists, 1,217 medical physicists, and 3,787 radiotherapy technologists. The Ministry of Health, Government of India has undertaken this proposed three-tier network in their National Cancer Control Programme through its Oncology Network (OncoNET) program (*Fig. 8*). The program envisages linking all the 27 regional cancer centers and 108 peripheral centers in the country through this OncoNET program, which would provide teleconsultation, cancer registration, tele-education, telepathology, and teleradiology.^24–27^

**Fig. 9.**
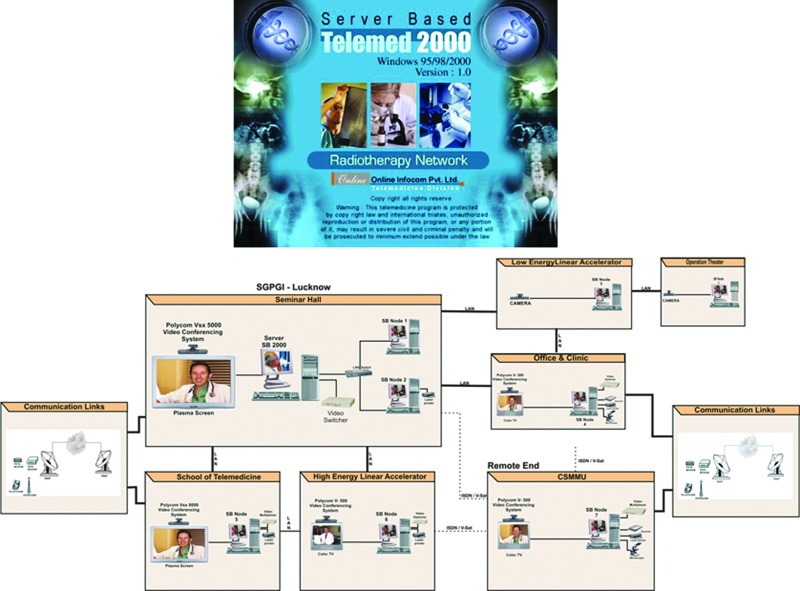
The teleradiotherapy network structure at Sanjay Gandhi Postgraduate Institute (SGPGI) of Medical Sciences, Lucknow, India, designed by the Online Telemedicine Research Institute, India. Color images available online at www.liebertpub.com/tmj

At the institution level, the Sanjay Gandhi Postgraduate Institute of Medical Sciences, Lucknow, India, was one of the first institutions to develop a teleradiotherapy network, in 2004. The project was funded by the Department of Science and Technology, Government of India, and the technical partner to the project was the Online Telemedicine Research Institute, Ahmedabad, India (*Fig. 9*).^16^ An audit carried out 2 years after the launching of the teleradiotherapy network and conducted between 2007 and 2009 showed that that the network provided a very effective media for teaching and training of the radiation oncology residents and other staff.^16^

## Application and Utility of a Teleradiotherapy Network

**Fig. 10.**
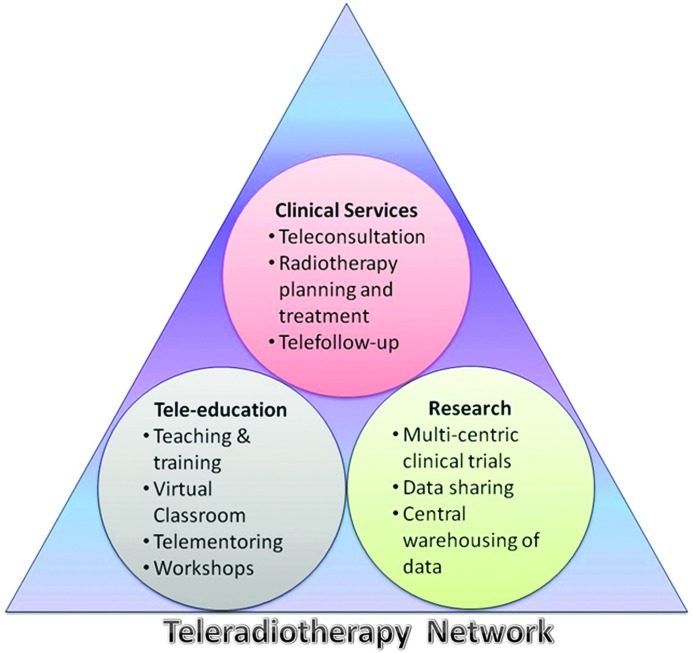
Teleradiotherapy network applications. Color images available online at www.liebertpub.com/tmj

A teleradiotherapy network could be an essential component of the tele-oncology applications along with a three-tier radiotherapy center concept using PRTCs, SRTCs, and TRTCs as detailed above. The utility of such a network could encompass various aspects of clinical services, teaching, training, and research (*Fig. 10*). These are as follows:

### Clinical Services

a. *Teleconsultation.* Patients attending the various departments of radiotherapy could be considered for a teleconsultation at a mutually agreed scheduled time wherein the treatment policies could be discussed based on the exchange of clinical details, radiological images, and other reports. Live consultation should be feasible in case the clinicians would like to see and interact with the patient.
b. *Radiation therapy planning for external beam radiotherapy and brachytherapy,* This could involve a detailed discussion on the radiation therapy delivery, treatment planning, plan evaluation, and online verification. In case the patient requires treatment using a linear accelerator with specialized techniques like three-dimensional conformal, intensity-modulated radiation therapy, stereotactic radiotherapy, or radiosurgery, which may not be feasible at other centers, these patients could be directly referred to the higher-level centers—SRTC or TRTC.
c. *Telefollow-up*. Follow-up makes up an important component of cancer management and needs to be conducted throughout the remaining life period of the cancer patient. Even though this would be carried out by the referring department, these could be discussed through the teleradiotherapy network with higher-level centers, in case the patient develops any untoward and unexpected problem both during and following the primary treatment.

### Teaching and Training

a. *Virtual classroom.* The teleradiotherapy linkage between the various departments of radiotherapy would help in developing a virtual classroom concept whereby one can formulate a common teaching program as per the predefined course syllabus for the postgraduate residents. The faculty from the various centers could be identified based on their expertise and interest, and these interactive teaching sessions could be beamed on to as many centers as permitted and supported by the hardware.^16^ Furthermore, this also opens up the avenues for international mentorship, especially for countries that would like to seek assistance from international faculty to assist in training their radiotherapy personnel. The International Atomic Energy Agency's PACT Programme through its Virtual University for Cancer Control Network has already initiated such a project for training radiation oncology personnel in Africa.^3,28–30^ This could be further expanded to countries lacking professional training programs.
b. *Teleconferencing and workshops.* The teleradiotherapy network among these centers would also help in teleconferencing and would enable the staff members to take part in workshops and other training programs, without the need of leaving their place of work. This is of specific importance as most of these centers are already running with limited staff, and absence for attending long training periods may severely affect the clinical workflow and patient care.

### Research

Research activities could be undertaken in a wide range of disease conditions predominant in the specified region with the network. The research activities could consist of:

a. *Multicenter trials.* These could be conducted among the participating institutions coordinated by a TRTC. These could be in form of Phase II/III randomized clinical trials targeted toward the most common cancers predominant in the country or region.
b. *Sharing of online data*. Data sharing, evaluation, and interim analysis could be feasible at the data processing centers of a TRTC. These could be made available to all centers following its processing and other statistical analysis. Periodic reviews could be conducted online through videoconferencing.
c. *Periodic review of the trials.* To ensure compliance, the progress of the clinical trials could be periodically reviewed by the group, and effective steps could be taken to ensure smooth conduct of the trials as designed in the protocols.

Moreover, all these activities could be linked to the national cancer control programs of the country for a wider cross-section of the radiation oncology and other oncology developmental activities and could be a cost-effective and realistic approach. The teleradiotherapy network thus could play a key role to address the problems related to radiotherapy infrastructure and human resources not only in LMICs but also in HICs.^31^

## Conclusions

The impending rise in cancer incidence during the next decades, especially in the LMICs, is a great challenge, and a multipronged approach is needed at various levels, at both national and international levels, to address it adequately. Radiotherapy, which is an important cancer treatment modality, is presently of limited availability in LMICs, which is a cause of major concern. The current approach in many LMICs of establishing very similar state-of-the-art-cancer centers in several parts of the country is not sustainable and in most cases also not feasible because of the large investments in equipment and human resources required. A more economic and effective approach is the establishment of a teleradiotherapy network where a main cancer center and a few other basic cancer treatment clinics including radiotherapy are already operational in a country. This could act as an effective means to improve the accessibility of radiotherapy services across a country or a region and allow for effective pooling and sharing of the limited infrastructure and staffing. The proposal of three-tier radiotherapy centers linked through networking provides a possible roadmap that allows patients to be treated closer to their homes and also allows them access to the state-of-the-art treatment modalities in radiotherapy as and when needed. The approach is cost-effective and deserves serious considerations by health planners within the context of national cancer control plans, at both national and international levels.
